# Clinical and laboratory characteristics of children with neurological presentations of COVID-19: a single-center experience

**DOI:** 10.25122/jml-2022-0184

**Published:** 2022-10

**Authors:** Nabeeha Najatee Akram, Basma Adel Ibrahim, Sabah Mohsin Ali, Wassan Nori

**Affiliations:** 1 Department of Pediatrics, Mustansiriyah University, Baghdad, Iraq; 2 Department of Obstetrics and Gynecology, Mustansiriyah University, Baghdad, Iraq

**Keywords:** COVID-19, neurological, pediatric, SARS-CoV-2

## Abstract

The study aimed to assess the frequency of neurological presentations of pediatric COVID-19 patients and compare the clinical and laboratory characteristics and the outcomes of those who presented with neurological complaints and those without complaints. A cross-sectional study enrolled 84 children diagnosed with COVID-19 at the emergency department over 12 months. All previously healthy children with a laboratory-confirmed diagnosis of COVID-19 were included in the study. The diagnosis of COVID-19 was made by positive PCR of a nasopharyngeal swab. Patients were divided into 2 groups: group 1 included COVID-19 patients with neurological complaints, and group 2 included COVID-19 patients with non-neurological complaints. Demographical, clinical, and laboratory characteristics were compared among groups. During the study period, 84 children aged 2 months-15years were diagnosed with COVID-19. Only 17 patients (20.2%) presented with new-onset neurological complaints. Seizure was the most common neurological complaint (58.8%), and febrile convulsion was the most frequent diagnosis of COVID-19 patients with neurological presentation (47.1%). C-reactive protein (CRP) and duration of hospitalization were higher in patients with neurological presentations, with P values of 0.002 and 0.001, respectively. All patients with neurological complaints survived the acute illness. Neurological symptoms were present in 20% of the COVID-19 pediatric patients, having higher CRP than patients with non-neurological presentations. CRP can be used as a reliable indicator for neurological symptoms in COVID-19 pediatric patients.

## INTRODUCTION

Since the beginning of the pandemic, infection with severe acute respiratory syndrome coronavirus 2 (SARS-CoV-2) has generated a variety of clinical symptoms. Despite the prevalence of respiratory symptoms in the pediatric age group, many children also exhibit neurological symptoms [1]. COVID-19 has been associated with various central and peripheral neurological injuries in adults and children, ranging from mild symptoms such as headache and anosmia to severe presentations such as stroke, seizure, and encephalopathy [2]. In a case series from Saudi Arabia, the neurological manifestations of COVID-19 in juvenile patients exhibited a wide range of clinical problems [3]. Uncertainty surrounds the extent to which the virus induced these neurological issues as opposed to the critical state, underlying degenerative disease, or iatrogenic effects of reusing medicines [4]. The incidence of neurological symptoms in adult COVID-19 patients varies greatly and approaches 100% [5, 6]. Although COVID-19-associated neurological problems in children are rather common, the published prevalence rates are significantly lower than adult prevalence rates [7, 8]. Due to a lack of adequate research, the frequency of neurological symptoms of COVID-19 in children is uncertain. A multi-center cohort study in the United States indicated that 40% of children with acute SARS-CoV-2 exhibited at least one neurological symptom [9]. Over the course of three months, Line et al. looked at the medical records of people with COVID-19 confirmed in the lab and found that 43% of them had neurological symptoms. LaRovere et al. reported the lowest rate, with only 22% of neurological involvement in COVID-19 children [10]. The reported prognosis for children with neurological manifestations of COVID-19 is excellent since nearly all have fully recovered [11]. In Iraq and other Arabic countries, there is a paucity of studies examining the neurological presentation of COVID-19 in children, with a single published case series from Saudi Arabia [3] and a case report from Iraq describing the neurological manifestation of COVID-19 as part of multisystemic inflammatory disease in childhood (MIS-C) [12]. Although a growing number of review articles and publications describe neurological manifestations of COVID-19 [11–13], its full impact on children under 18 years old is yet unknown [14]. This research intended to determine the prevalence and types of neurological symptoms in COVID-19-infected children. The secondary objective was to discover potential predictors of neurological symptoms.

## Material and methods

A descriptive, cross-sectional, single-center study was conducted over 12 months from 1^st^ April 2020 to 31^st^ March at the emergency department of a central child teaching hospital in Baghdad, Iraq.

The inclusion criteria:

- Children with a laboratory-confirmed diagnosis of COVID-19 confirmed by positive polymerase chain reaction (PCR) of a nasopharyngeal swab;
- Previously healthy children (an absence of reported underlying chronic diseases or comorbidities and taking no prescription medications).

We excluded cases with underlying chronic illness or comorbidities and those who refused to give informed consent. All patients had a full neurological assessment by a pediatric neurologist. Accordingly, they were sub-divided into group 1, which included cases with new-onset neurological complaints, and group 2, which included those without neurological complaints.

The demographical, clinical (type of neurological complaints, diagnosis, hospitalization period, and the outcome), and patients laboratory characteristics (C-reactive protein (CRP), total white blood cell, absolute lymphocyte, and absolute neutrophile count, packed cell volume (PCV), and platelets count) were collected and compared for a possible correlation.

Families gave informed consent after briefing about the study's aim. The statistical analysis was carried out using SPSS software version 26. The mean and standard deviation of data with a normal distribution were provided, and the student's T-test was used for comparison. The Mann-Whitney U test was used to assess data with non-normal distributions, which were reported as median and range. The Chi-square test was used to examine categorical variables expressed as numbers and percentages. A statistically significant result was defined as a p-value of less than 0.05.

## Results

**Figure 1:**
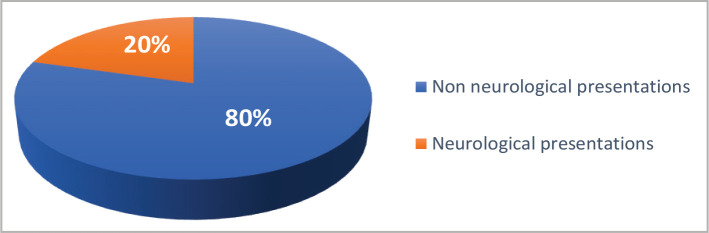
The frequency of neurological presentations in children hospitalized with COVID-19.

During the study period, 84 children (aged 2 months to 15 years) were diagnosed with COVID-19 at the central child teaching hospital and enrolled in the study. Males outnumbered females (54% *versus* 46%). Only 17(20.2%) patients presented with neurological complaints on admission to the hospital and/or during hospitalization, while 67(79.8%) patients had non-neurological presentations (Figure 1).

In patients with neurological complaints, seizure was the most common neurological complaint seen in 58.8% of patients, followed by headache (47.1%), acute flaccid paralysis, and disturbed level of consciousness (11.8%) (Table 1).

**Table 1:** Types of neurological presentations in COVID-19 patients (n=17).

Neurological complaints	Frequency (%) *
Seizure	10 (58.8%)
Headache	6 (47.1%)
Acute flaccid paralysis	2 (11.8%)
Disturbed level of consciousness	2 (11.8%)

* – Patients can have more than one complaint.

Febrile convulsion was the most frequent neurological diagnosis of COVID-19 patients (47.1%), followed by encephalitis (17.6%) (Table 2). Two cases presented initially with fever and seizure, but during hospitalization, other neurological complaints occurred (one of them developed ataxia and the other hemiparesis), and based on CSF analysis and brain imaging, they were diagnosed with acute demyelinating encephalomyelitis (ADEM).

**Table 2:** Diagnoses of COVID-19 pediatric patients who presented with neurological complaints (n=17).

Diagnosis	Frequency (%)
Febrile seizure	8 (47.1%)
Encephalitis	3 (17.6%)
Guillain barre Syndrome (GBS)	2 (11.8%)
Viral meningitis	2 (11.8%)
Acute demyelinating encephalomyelitis (ADEM)	2 (11.8%)

Duration of hospitalization (p=0.001) and C-reactive protein (CRP) (p=0.002) were higher in patients with neurological presentations. On the other hand, the age and gender of patients, leukocyte counts, absolute neutrophil counts, and absolute lymphocyte counts were not statistically associated with the neurological presentations, as shown in Table 3.

**Table 3:** Association of patient's characteristics with patient's presentation.

Variables	Neurological presentations of COVID-19		P-value
	Yes (n=17)	No (n=67)	
**Age (years)**			
Mean±SD	7.6±3.7	5.3±4.6	0.67 ^†^
**Gender**			
Male	13 (76.5%)	52 (77.6%)	0.92 *
Female	4 (23.5%)	15 (22.4%)	
**Duration of hospital stay (days)**			
Mean±SD	5.76±5.5	3.15±1.5	0.001 ^†^
**Lymphocytes×10^3^/ml**			
Median (range)	2.3 (0.2–5.9)	1.6 (0.4–7)	0.72 ^‡^
**Neutrophil×10^3^/ml**			
Median (range)	3.4 (2.1–13.1)	4.6 (0.9–15.9)	0.65 ^‡^
**Platelets×10^3^/ml**			
Median (range)	259 (169–515)	250 (8–515)	0.94 ^‡^
**Total WBC×10^3^/ml**			
Median (range)	7.7 (4.9–21.9)	7.8 (1.07–23.8)	0.59 ^‡^
**CRP mg/dl**			
Median (range)	112 (0–168)	30 (0–146)	0.002 ^‡^
**PCV**			
Median (range)	33 (17–37)	30.9 (18–37)	0.58 ^‡^

* – Chi-square test; ^†^ – Student t-test; ^‡^ – Mann Whitney U test; CRP – C-reactive protein; PCV – packed cell volume; WBC – white blood cell.

In what concerns patients' outcome, only four (4.8%) out of the 84 children enrolled in this study died during hospitalization, and none showed new-onset neurological symptoms.

## Discussion

Since the beginning of the epidemic, physicians have become more aware of neurological symptoms in COVID-19 patients. It has been suggested that neurologists should be involved in the care of COVID-19 patients [15]. It is very important to find out if SARS-CoV2 is linked to neurological symptoms, as this could lead to significant morbidity and mortality [4]. In this study, 20.2% of the children with COVID-19 had new neurological symptoms, which agrees with a USA study reporting that 22% of SARS-CoV-2 hospitalized children had neurological manifestations [10]. However, this frequency is lower than that reported by two previous studies by Lin et al. (43%) [2] and Fink et al. (40%) [9]. In the current study, the most common neurological symptom in COVID-19 children was a seizure (58.8%). This disagrees with previous studies that reported headache [9] and fatigue [10] as the most common neurological symptom. The difference in the frequency and type of neurological manifestation in this study can be attributed to the lower age of patients compared to previous studies. This could make it hard for patients and their families to describe some neurological symptoms. Under-reporting of headaches in children is common [16, 17]. Even though the rate of neurologic manifestations in pediatric patients with COVID-19 in this study was low, raising clinical awareness regarding neurological complications of COVID-19 to recognize and treat patients early and adequately is vital. For some neurological complications of COVID-19, like GBS and ADEM, a rapid diagnosis and immediate therapy are vital [18]. Concerning the diagnoses, most (47.1%) cases with a neurological presentation were labeled as having febrile convulsions when sent home. This fits with other studies and reviews that seizures, especially simple febrile seizures, are the most common neurological sign of a coronavirus infection [19].

However, it is worth mentioning that although all these patients underwent cerebral spinal fluid (CSF) testing, which was normal in analysis and negative for bacterial pathogen culture, PCR for SARS-COV2 in CSF was not done in this study due to the unavailability of the test in our center. In this study, the mean duration of hospital stays for neurological cases (5.76±5.5) was significantly higher than those with non-neurological presentation days, and this agrees with the results by Fink et al. [9]. Nevertheless, it was contrary to LaRovere et al., who found that the duration of intensive care unit, not hospitalization, was significantly related to the presence of neurological manifestation in COVID-19 [10]. This can be attributed to different clinical diagnoses compared with mild diagnoses in the current study, in which (47.1%) were diagnosed with febrile convulsion, which usually requires a short hospital stay [20]. Although patients with neurological manifestations are older than those with non-neurological complaints, the age of pediatric COVID-19 with neurological presentations did not differ significantly from children with non-neurological ones. This is contrary to results reported in previous studies. Fink et al. found that COVID-19 children who present with neurological symptoms tend to be significantly older [9]. The authors believe that this could be the result of many adolescent children, especially those aged ≥16 years, receiving medical care in adult hospitals in Iraq and, as a result missing many cases of neurological presentation of adolescents COVID-19.

In this study, none of the blood indices was significantly related to the presence of neurological manifestations in COVID-19 children, so the above indices do not appear to be reliable indicators of neurological manifestations of pediatric COVID-19, and this agrees with previous studies which document that hospitalized COVID-19 pediatric patients regardless of the type of presentation had no significant changes in laboratory parameters [21, 22]. The cause may be attributed to the non-severe neurological diagnosis carried by about half of the patients. Studies in adults proved that the median CRP value correlates with the severity of COVID-19 and is an independent predictor of mortality [23]. To our best knowledge, no previous study compared blood indices and inflammatory markers in COVID-19 children according to the presence or absence of neurological complaints. Herein CRP was significantly higher in patients who presented with neurological symptoms. CRP is an inexpensive, widely available test used in COVID-19 pediatric patients for early recognition of neurological presentations for early and adequate treatment. All patients with neurological complaints survived the acute illness, which agrees with previous studies [11]. This represents one major difference from the adult population in which the prognosis of COVID-19 cases with neurological presentation had shown a higher risk of mortality regardless of the type of neurological pathology. The risk is aggravated by older age and higher inflammatory markers [24, 25]. The current study had several limitations. First, the small sample size and the fact that it was conducted at a single center in Baghdad-Iraq. Second, after hospital discharge, follow-up was not done, so long-term or delayed neurological complications of COVID-19 were not reported. Third, some neurological complaints, like headaches and fatigue, are subjective, and underreporting is possible. Finally, this study did not conduct PCR for SARS-COV2 in CSF due to unavailability.

## Conclusion

In the pediatric age group, neurological symptoms were present in 20% of COVID-19 patients. Higher CRP levels in COVID-19 pediatric patients are significantly associated with neurological complaints since CRP is an inexpensive, widely available test, so it can be used in COVID-19 pediatric patients for early recognition of neurological presentations to treat patients early and adequately.

## Personal thanks

We would like to thank Mustansiriyah University, Baghdad-Iraq, for its support in the current study.

## References

[1] Jorden MA, Rudman SL, Villarino E, CDC COVID-19 Response Team (2020). Evidence for Limited Early Spread of COVID-19 Within the United States, January-February 2020. MMWR Morb Mortal Wkly Rep.

[2] Lin JE, Asfour A, Sewell TB, Hooe B (2021). Neurological issues in children with COVID-19. Neuroscience letters.

[3] Aljomah L, Almedlej S, Baarmah D, Altwaijri W (2021). Pediatrics COVID-19 and neurological manifestations: Single tertiary centre experience. E Neurological Sci.

[4] Ellul M, Varatharaj A, Nicholson TR, Pollak TA (2020). Defining causality in COVID-19 and neurological disorders. Journal of Neurology, Neurosurgery & Psychiatry.

[5] Pezzini A, Padovani A (2020). Lifting the mask on neurological manifestations of COVID-19. Nature reviews Neurology.

[6] Sharifian-Dorche M, Huot P, Osherov M, Wen D (2020). Neurological complications of coronavirus infection; a comparative review and lessons learned during the COVID-19 pandemic. Journal of the neurological sciences.

[7] Panda PK, Sharawat IK, Panda P, Natarajan V (2021). Neurological Complications of SARS-CoV-2 Infection in Children: A Systematic Review and Meta-Analysis. Journal of tropical pediatrics.

[8] Ranabothu S, Onteddu S, Nalleballe K, Dandu V (2020). Spectrum of COVID-19 in children. Acta Paediatrica.

[9] Fink EL, Robertson CL, Wainwright MS, Roa JD (2022). Prevalence and risk factors of neurologic manifestations in hospitalized children diagnosed with acute SARS-CoV-2 or MIS-C. Pediatric neurology.

[10] LaRovere KL, Riggs BJ, Poussaint TY, Young CC (2021). Neurologic involvement in children and adolescents hospitalized in the United States for COVID-19 or multisystem inflammatory syndrome. JAMA neurology.

[11] Principi N, Esposito S (2021). Are we sure that the neurological impact of COVID 19 in childhood has not been underestimated?. Italian journal of pediatrics.

[12] Akram NN, Nori W, Al Qaissi K (2021). Multi-Systemic Inflammatory Syndrome in Childhood with Neurological Presentation: A Case Report. AlQalam Journal of Medical and Applied Sciences.

[13] Akram NN, Nori W, Al Qaissi KW, Abdulrahman Hadi BA (2021). Multisystemic inflammatory syndrome in childhood (MIS-C): A review article. The Journal of the Pakistan Medical Association.

[14] Leonardi M, Padovani A, McArthur JC (2020). Neurological manifestations associated with COVID-19: a review and a call for action. Journal of neurology.

[15] Liu K, Pan A, Xiao Z, Xu X (2020). Neurological manifestations of the coronavirus (SARS-CoV-2) pandemic 2019-2020. Journal of Neurology, Neurosurgery & Psychiatry.

[16] Lundqvist C, Clench-Aas J, Hofoss D, Bartonova A (2006). Self-reported headache in schoolchildren: Parents underestimate their children's headaches. Acta Paediatrica.

[17] Wöber-Bingöl Ç (2013). Epidemiology of migraine and headache in children and adolescents. Current pain and headache reports.

[18] Berlit P, Bösel J, Gahn G, Isenmann S (2020). "Neurological manifestations of COVID-19"-guideline of the German society of neurology. Neurological Research and Practice.

[19] Singer TG, Evankovich KD, Fisher K, Demmler-Harrison GJ, Risen SR (2021). Coronavirus Infections in the Nervous System of Children: A Scoping Review Making the Case for Long-Term Neurodevelopmental Surveillance. Pediatric Neurology.

[20] Kwong KL, Tong KS, So KT (2003). Management of febrile convulsion: scene in a regional hospital. Hong Kong Med J.

[21] Akram NN (2022). Characteristics and Outcome of Hospitalized Children with COVID-19: A Single Center Experience. Indian Journal of Forensic Medicine & Toxicology.

[22] Henry BM, Benoit SW, de Oliveira MHS, Hsieh WC (2020). Laboratory abnormalities in children with mild and severe coronavirus disease 2019 (COVID-19): A pooled analysis and review. Clinical biochemistry.

[23] Sharifpour M, Rangaraju S, Liu M, Alabyad D (2020). C-Reactive protein as a prognostic indicator in hospitalized patients with COVID-19. PloS one.

[24] Deeb A, Kumar PC, Sakrani N, Trehan RK, Papinenei VR (2021). Neurological Presentations of COVID-19: Characteristic Features in a Case Series of Hospitalized Patients from Abu Dhabi, UAE. BioMed research international.

[25] Leven Y, Bösel J (2021). Neurological manifestations of COVID-19-an approach to categories of pathology. Neurological Research and Practice.

